# Bilateral lower extremity inflammatory lymphedema after an ultramarathon

**DOI:** 10.1016/j.jdcr.2023.08.002

**Published:** 2023-08-09

**Authors:** William H. Robinson, Hal B. Willardson, Nathaniel S. Nye

**Affiliations:** a Naval Hospital Camp Pendleton, Oceanside, California; b Dermatology Clinic, 673rd Medical Group, Joint Base Elmendorf-Richardson, Anchorage, Alaska; c Sports Medicine Clinic, Fort Belvoir Community Hospital, Fort Belvoir, Virginia

**Keywords:** exercise, lymphatic, lymphedema, ultramarathon, vasculitis

## Introduction

Although the strain of long-distance running on the skin and cardiorespiratory system is well recognized,^1^^,^^2^ strain on the venous and lymphatic systems is often overlooked. Here, we document a case of bilateral lower extremity inflammatory lymphedema (BLEIL) after an ultramarathon race in an otherwise healthy male endurance athlete.

## Case report

A 54-year-old male ultramarathoner presented with severe swelling, redness, and pain in bilateral lower extremities. Symptoms began a few hours after finishing a 100-mile trail-running race in eastern Texas. The race occurred in February, with ambient temperatures ranging from 44 to 76 °F. The swelling progressively worsened, and he presented to the clinic approximately 3 days after finishing. He reported having run almost continuously for >30 hours but denied any specific injury. He denied any changes in urine color, fever, severe/focal pain, or numbness/tingling. He reported running 3 prior races of 100+ miles but denied any history of similar symptoms. His medical and surgical histories were unremarkable.

**Fig 1:**
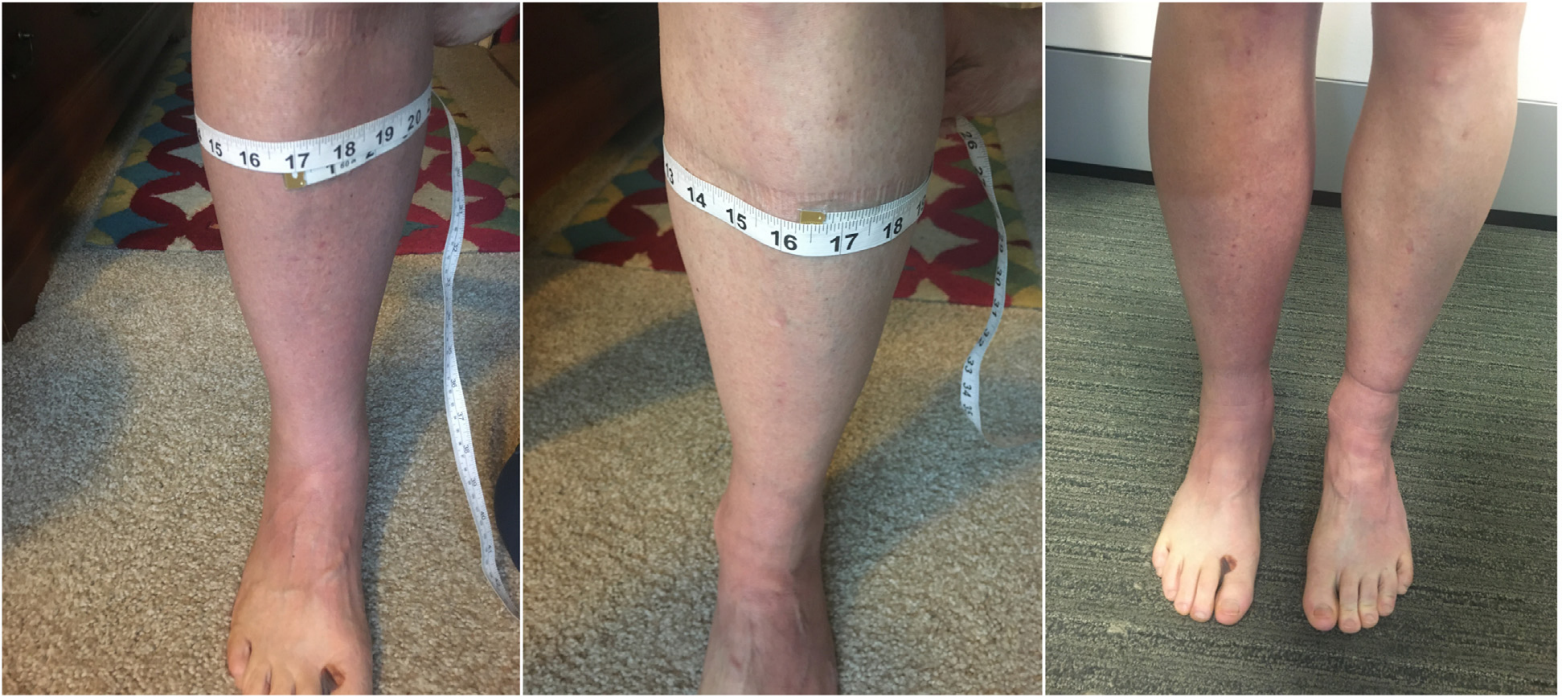
Photographs taken of the patient’s legs on day 5 after completion of the 100-mile ultramarathon race. Mild confluent erythema and pitting edema, with lack of petechiae or purpura.

The patient arrived in a wheelchair but transitioned to the examination table unassisted. His vital signs were normal, and he was in no distress. Upon inspection, prominent 4+ pitting edemas and moderate diffuse erythema and warmth were noted from the knees to the toes bilaterally (Fig 1). There was no purpura. The knees, ankles, and feet were nontender, and there was only mild tenderness to palpation of the lower leg muscular compartments. There was mild pain on passive ankle dorsiflexion and plantarflexion. Strength testing revealed mild weakness (4+/5) in ankle dorsiflexion and plantarflexion. Neurovascular examination was unremarkable.

Initial laboratory testing revealed a serum creatine kinase (CK) level of 5800 U/L and urinalysis positive for protein (30 mg/dL). Urine was negative for hemoglobin, and only rare red blood cells were present in sediment. A basic metabolic panel and complete blood cell count were completely normal.

Bilateral tibia/fibula x-rays were normal. Ultrasound of the lower legs showed widespread thickening of the subdermal adipose layer with prominent streaky anechoic signal interspersed among the hypoechoic fat lobules bilaterally and mild anechoic tenosynovial effusion of the anterior, medial, and lateral ankle tendons. Muscles in all 4 compartments demonstrated normal architecture throughout.

A clinical diagnosis of BLEIL was made. HyperCKemia versus possible mild exertional rhabdomyolysis was considered, although this was unlikely to be the cause of his profuse subcutaneous edema and erythema. His mild proteinuria was considered benign and due to the extreme exertion.^3^ He was released to home with instructions to compress and elevate the legs for 72 hours. He was instructed to perform ankle pumps and calf raises to increase lymphatic drainage.

The following day (day 4 after the race), serum CK level was 1964 U/L. The patient followed up in the clinic on day 9 after the race. He experienced significant improvement of symptoms but continued to experience mild pain and swelling (right > left). He was now ambulating, although still with slightly antalgic gait. Examination revealed persistent but markedly improved erythema and edema of bilateral legs and improved strength at the ankles. At the 1-month follow-up, symptoms and examination findings had resolved, and CK and urinalysis had normalized. He was then cleared to resume light conditioning, progressing gradually as tolerated, with follow-up as needed.

## Discussion

BLEIL was first described in 2015 among military basic trainees after prolonged periods of standing or marching.^4^ The physical exertion, well beyond accustomed levels, resulted in decompensated strain on the venous and lymphatic systems. Clinically, the condition resembles cellulitis of bilateral lower legs, ankles, and feet, showing a rapid onset of diffuse tenderness, erythema, warmth, and edema. The pathophysiology results from prolonged venous congestion from the upright position, leading to interstitial edema, leakage of proteins and cytokines into the interstitial space, overload of the lymphatic system, and a subsequent inflammatory leukocytoclastic vasculitis.^4^^,^^5^

Endurance running has been shown to induce strain and subsequent adaptations on the vascular and lymphatic systems. A recent study compared venous and lymphatic system characteristics in habitual long-distance runners 5 days after running a marathon with those of sedentary controls.^6^ The marathoners demonstrated increased capillary and venous flow capacity at rest and greater increase in venous and lymphatic flow after exercise (via Doppler and plethysmography studies).

On initial evaluation, the differential diagnosis often includes contact dermatitis, cellulitis, and rhabdomyolysis.^2^ The bilateral symmetric distribution, diffuse erythema and prominent edema, lack of fever, and normal or mildly elevated CK levels are important clues to the diagnosis of BLEIL. Exercise-induced vasculitis (EIV), also known as “Golfer’s vasculitis” or “Disney rash,” can also present with erythema and edema of the lower legs after prolonged activity.^4^^,^^7^, ^8^, ^9^, ^10^ However, BLEIL and EIV differ in a few key ways. First, EIV presents with very distinct purpuric lesions interspersed among patchy erythema and mild edema, typically sparing the skin covered by clothing, such as socks.^10^ In contrast, BLEIL presents with bilateral diffuse and confluent erythema and a more significant and painful pitting edema of the lower extremities and lacks any purpuric or petechial lesions.^4^^,^^5^ Also, EIV (but apparently not BLEIL) is associated with exertion in hot weather.^7^, ^8^, ^9^ Additionally, there are histologic differences. Biopsy specimens of patients with EIV reveal a classic leukocytoclastic vasculitis, with predominance of neutrophils and karryorhectic debris around the small venules of the superficial dermis, which correspond to the palpable purpuric lesions described clinically.^7^, ^8^, ^9^ Conversely, biopsy specimens of BLEIL reveal a mixed perivascular and perieccrine infiltrate concentrated in the deep reticular dermis.^5^

In conclusion, BLEIL is a self-limiting condition that is often misdiagnosed and overtreated and must be distinguished from other similar conditions. When BLEIL is diagnosed accurately, unnecessary medical workups, hospitalizations, and antibiotics can be avoided. Treatment of BLEIL should focus on elevation of the lower extremities for 48 to 72 hours, progressing to ambulation as tolerated, and “muscle pump” exercises to increase venous and lymphatic return. Patients can be reassured that their symptoms will improve with conservative treatment in 7 to 14 days. This may reduce patient anxiety as well as the risks inherent to medical testing and treatments. With the rising popularity of endurance sports such as ultrarunning, a keen awareness of the illnesses and injuries that may result is critical.

## Conflicts of interest

None disclosed.
